# An investigation of the correlation between IL-8, IL-10 and nutritional proteins in patients with sleep disorders in chronic obstructive pulmonary disease

**DOI:** 10.5937/jomb0-54163

**Published:** 2025-09-05

**Authors:** Mei Liang, Mei Zhou, Xiaofeng Fu, Qianyun Zhou, Liang Li

**Affiliations:** 1 The People's Hospital of Yubei District, Department of Respiratory and Critical Care Medicine, Chongqing, China; 2 The People's Hospital of Yubei District, Department of Laboratory Medicine, Chongqing, China

**Keywords:** chronic obstructive pulmonary disease, immune microenvironment, interleukin-10, interleukin-8, nutritional proteins, sleep disorder, hronična opstruktivna bolest pluća, imunološko mikrookruženje, interleukin-10, interleukin-8, nutritivni proteini, poremećaj spavanja

## Abstract

**Background:**

Chronic obstructive pulmonary disease (COPD) is one of the most common respiratory diseases worldwide, with an increasing incidence in recent years. In this study, we analysed the relationship between interleukin-8 (IL-8), interleukin-10 and COPD to provide a reference for clinical diagnosis and treatment in the future.

**Methods:**

A randomised controlled trial was conducted on 56 COPD patients and 56 concurrent healthy volunteers who visited our hospital from March 2022 to December 2022. Among them, COPD patients served as the research group, and healthy volunteers served as the control group. To compare the IL-8 and IL-10 of the two groups and to analyse the relationship between IL-8, IL-10 and lung function, nutrient proteins, clinical efficacy and prognosis of the research group.

**Results:**

IL-8 was higher in the study group than in the control group and was negatively correlated with lung function indices and nutrient proteins (P<0.05). IL-10 in the research group was lower than in the control group, and there was a positive correlation with lung function indexes and nutritional proteins (P<0.05). After treatment, IL-8 was lower, and IL-10 was higher in the research group (P<0.05). In addition, IL-8 and IL-10 in the research group demonstrated excellent assessment of COPD occurrence, sleep disturbance, and prognostic recurrence.

**Conclusions:**

IL-8 and IL-10 not only directly participate in the occurrence of COPD by affecting the human immune microcirculation but also accelerate the progression of COPD by causing malnutrition.

## Abstract (sr)

**Uvod:**

Hronična opstruktivna bolest pluća (HOBP) je jedna od najčešćih respiratornih bolesti u svetu, sa rastućom incidencijom u poslednjim godinama. U ovoj studiji smo analizirali odnos između interleukina-8 (IL-8), interleukina-10 i HOBP-a kako bismo pružili referentne podatke za buduću kliničku dijagnostiku i lečenje.

**Metode:**

Sprovedeno je randomizovano kontrolisano istraživanje na 56 pacijenata sa HOBP-om i 56 zdravih volontera koji su posetili našu bolnicu od marta 2022. do decembra 2022. Među njima, pacijenti sa HOBP-om su činili grupu nad kojom je sprovedeno istraživanje, dok su zdravi volonteri činili kontrolnu grupu. Cilj je bio da se uporede nivoi IL-8 i IL-10 u obe grupe, kao i da se analizira odnos između IL-8, IL-10 i funkcije pluća, nutritivnih proteina, kliničke efikasnosti i kao i prognoza grupe nad kojom je vršeno istraživanje.

## Introduction

Chronic obstructive pulmonary disease (COPD), a complex respiratory disease characterised by progressive decline in lung function, chronic airway inflammation, and decreased quality of life, is a common and frequently occurring disease with high mortality [1]. COPD shows a prevalence of about 8.6% in China, of which 13.6% are over 40 years old, and afflicts approximately 100 million cases, which is considered to be the third leading cause of death in China [2]. Glucocorticoids remain the primary clinical treatment for COPD, and the mechanism lies in binding to hormone receptors, inhibiting the activity of transcription factors that can promote the expression of inflammatory genes, and playing an anti-inflammatory role in reversing the progression of COPD [3]. Clinical studies have shown that the typical clinical manifestations of COPD include sudden, recurrent dyspnea, which is particularly prevalent at night [4]. This has led to severe sleep disorders in most COPD patients, and clinical studies have shown that about 40-50% of COPD patients have sleep disorders [4]. Sleep is a critical process in human life activities, and good sleep is directly and closely related to the nutritional state of the human body, as well as its effects on immune function and metabolic capacity. The occurrence of sleep disorders in COPD may not only exacerbate the progression of COPD but also promote the occurrence of complications in other organs, which threatens the health of patients [5]. Therefore, we need an effective and accurate clinical indicator for assessing COPD patients' sleep disorders to develop interventions.

Interleukins (ILs) are lymphokines that regulate leukocytes and immune cells in the human body and are essential substances in inflammation and immune responses [6]. Among them, IL-8 is a chemotactic factor that can activate neutrophils to reach the inflammatory site and play a role. IL-8 in sputum is highly correlated with the composition and diversity of the microbiota [7]. IL-10 is an anti-inflammatory factor that effectively prevents the synthesis of proinflammatory factors and IL-8 in macrophages and synovial cells, and the decrease of its level is closely related to the progression of COPD [8]. Recently, a report by Jesus FR et al. [9] mentioned that among the many inflammatory factors in the human body, IL-8 and IL-10 will probably be important potential markers of COPD.

We hypothesised that IL-8 and IL-10 may potentially coordinate in COPD and influence the occurrence of sleep disorders in COPD patients, but no study has yet been conducted to corroborate our conjecture. Therefore, the present study will analyse the clinical significance of IL-8 and IL-10 in patients with COPD sleep disorders and further analyse the relationship between the two and the nutritional status of the patients to provide new references and guidelines for future clinical diagnosis and treatment of COPD sleep disorders.

## Materials and methods

### Study population

The sample size required for this study was calculated using the PASS software (NCSS, Switzerland) with =0.5, which showed that a minimum of 46 study subjects were required in each group. A randomised controlled trial was conducted on 56 COPD patients and 56 concurrent healthy volunteers who visited our hospital from March 2022 to December 2022. Among them, COPD patients served as the research group, and healthy volunteers served as the control group. This study has been approved by the Ethics Committee of our hospital, No. 2020(33), and all the subjects have signed an informed consent form.

### Eligibility and exclusion criteria

Inclusion criteria: Conforming to CODP diagnostic guidelines [10]; forced expiratory volume in the first second (FEV1)/forced vital capacity (FVC) <70% and FEV1%pred <80% in the lung function tests; no use of antibiotics, immunosuppressants, cytotoxic agents, or other drugs affecting respiratory microorganisms one month before enrollment. Exclusion criteria: Severe hepatic and renal insufficiency; active tuberculosis and hemoptysis; bronchiectasis, pulmonary fibrosis, asthma, or other lung diseases; tumours; myocardial infarction attacks within one year, severe heart failure within two years, or arrhythmia requiring drug control; use of oral antibiotics or immunosuppressants in the last month; severe mental disorders, or inability to complete this study independently.

### Treatment methods

Patients in the research group received symptomatic treatments such as oxygen inhalation, bronchodilation, anti-infection, anti-inflammation, and maintenance of water-electrolyte balance after admission. Antibiotics were given according to the results of the drug susceptibility test. In addition, inhalation therapy with salmeterol/fluticasone propionate powder was administered at a dose of 50 μg/250 μg once a day for 2 weeks.

### Efficacy evaluation

We evaluated efficacy by referring to COPD treatment guidelines [11]: Marked effectiveness refers to relieved clinical symptoms and disappearance of lung rales after treatment; the improvement of clinical symptoms and a significant reduction in lung rales are considered effective; ineffectiveness means no improvement in symptoms and no reduction in lung rales.

### Assessment of sleep disorders

Patients' sleep quality was assessed using the Pittsburgh sleep quality index (PSQI) (Zitser et al. [12], with a total score of 21, and a score result of >5 for 3 consecutive d was considered to be the presence of a sleep disorder.

### Prognostic follow-up

A one-year prognostic follow-up was conducted on all COPD patients in regular follow-ups, with an interval of no more than 2 months. The recurrence of COPD in patients within one year of prognosis was recorded.

### Sample collection and testing

At admission, FEV1 and FVC were detected in both groups with a pulmonary function tester. Blood samples of the control group at admission and those of the research group at admission and after treatment were collected to measure IL-8 and IL-10 levels following the instructions of enzyme-linked immunosorbent assay kits (Eimage Technology Co., Ltd, China). Blood samples were collected in procoagulant tubes, left to stand for 30 minutes at room temperature, and then centrifuged (3000 rpm/min) for 10 minutes to obtain serum for subsequent testing. Albumin (ALB), haemoglobin (Hb), and prealbumin (PA) were measured with an automatic biochemical analyser (BS-830, Mindray, China)

### Endpoints

(1) Differences in IL-8, IL-10, lung function, and nutritional proteins between the research and control groups were analysed. (2) The diagnostic value of IL-8 versus IL-10 for COPD was analysed by receiver operating characteristic (ROC) curves, and the effectiveness of diagnosis was rated by area under the curve (AUC). (3) Pearson's correlation coefficient analysed Correlations between IL-8, IL-10 and lung function, nutritional proteins, and clinical efficacy.

### Statistical analyses

Statistical analysis was performed using SPSS26.0 (IBM, USA). The (x̄±s) were used to describe continuous variables statistically, and independent sample t-tests (normal distribution) or Mann-Whitney U tests (non-normal distribution) were used for comparisons. The comparison of count data [n(%)] used chi-square tests. The diagnostic value was analysed by ROC curves, with the AUC closer to 1 suggesting better diagnostic effects. Pearson correlation coefficients analysed correlations. A minimum significance threshold of P<0.05 was used.

## Results

### There was no difference in clinical data between the two study groups

As shown in Table 1, statistical analysis of the age, gender, and course of COPD between the two groups showed no significant difference (P>0.05), suggesting comparability.

**Table 1:** Comparison of clinical data.

Groups (n=56)	n	Male	Female	Age	Duration of<br>disease (years)	Body mass index<br>(kg/m^2^)
Control	56	39 (69.64)	17 (30.36)	67.79±5.67	-	21.50±1.72
Research	56	34 (60.71)	22 (39.29)	66.79±5.12	2.38±0.95	21.99±1.89
χ^2^ (t)		0.984	0.980	-	1.436	
P		0.321	0.329	-	0.154	

### IL-8 was higher in the research group than in the control group, while IL-10 was lower than in the control group

**Figure 1:**
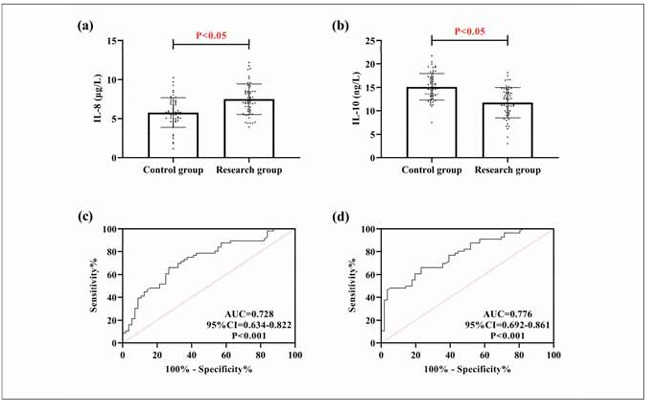
Comparison of IL-8 and IL-10. (a) comparison of IL-8 in research and control groups. (b) comparison of IL-10 in research and control groups. (c) ROC curve for IL-8 diagnosis of COPD occurrence. (d) ROC curve for IL-10 diagnosis of COPD occurrence. Receiver operating characteristic, ROC.

As shown in Figure 1, the research group showed higher IL-8 and lower IL-10 than the control group (P<0.05). Through ROC curve analysis, it can be seen that when IL-8>6.88 μg/L, the sensitivity and specificity for diagnosing COPD were 66.07% and 73.21%, respectively (P<0.05); when IL-10 was less than 13.24 ng/L, its diagnostic sensitivity was 66.07% and the specificity was 76.79% (P<0.05). It is suggested that both IL-8 and IL-10 have good diagnostic efficacy for COPD.

## IL-8 and IL-10 show excellent diagnostic value for sleep disorders

**Figure 2:**
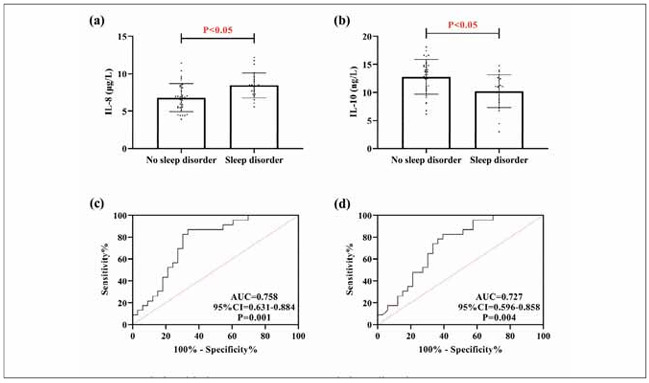
Relationship between IL-8, IL-10 and sleep disorders. (a) comparison of IL-8 in patients with and without sleep disorders. (b) comparison of IL-10 in patients with and without sleep disorders. (c) ROC curves for IL-8 diagnosis of sleep disorders occurring in COPD patients. (d) ROC curves for IL-10 diagnosis of sleep disorders occurring in COPD patients.

As shown in Figure 2, 23 out of 56 COPD patients had sleep disorders, and the incidence of sleep disorders was 41.07%. Upon comparison, it was seen that IL-8 was higher in patients with sleep disorders than in patients without sleep disorders, while IL-10 was lower than in patients without sleep disorders (P<0.05).The ROC curves showed that the sensitivity of diagnosing the occurrence of sleep disorders in patients with COPD was 86.96%. The specificity was 66.67% when IL-8>7.06 μg/L (P<0.05), and the specificity of diagnosing the occurrence of sleep disorders in patients with COPD was 86.96% and 66.67% when IL-10<12.68ng/L, the sensitivity of diagnosing sleep disorder in COPD patients was 82.61%. The specificity was 60.61% (P<0.05).

### Relationship between IL-8, IL-10, and lung function

**Figure 3:**
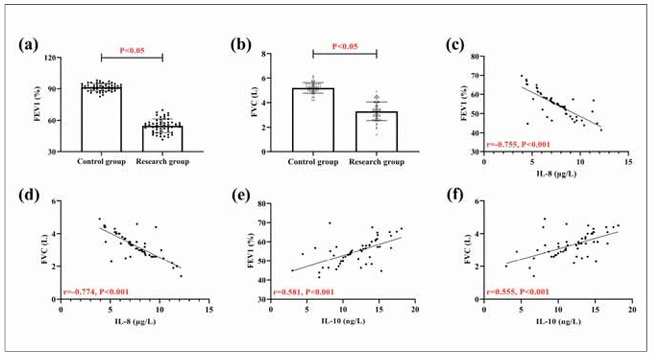
Relationship between IL-8, IL-10, and lung function. (a) comparison of FEV1 in research and control groups. (b) comparison of FVC in research and control groups. (c) correlation of IL8 and FEV1. (d) correlation of IL-8 and FVC. (e) correlation of IL-10 and FEV1. (f) Correlation of IL-10 and FVC. Forced expiratory volume in the first second, FEV1; Forced vital capacity, FVC.

As can be seen from Figure 3, FEV1 and FVC were lower in the research group than in the control group (P<0.05). According to Pearson correlation coefficient analysis, IL-8 in the research group was negatively correlated with FEV1 and FVC (P<0.05), while IL-10 was positively correlated with them (P<0.05), suggesting that both IL-8 and IL-10 are closely related to patients' lung function.

### Correlation of IL-8 and IL-10 with nutritional proteins

**Figure 4:**
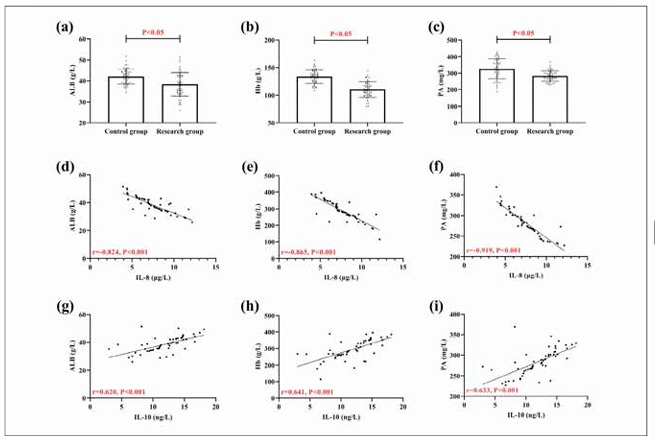
Correlation of IL-8 and IL-10 with nutritional proteins. (a) comparison of ALB in research and control groups. (b) comparison of Hb in research and control groups. (c) comparison of PA in research and control groups. (d) correlation of IL-8 and ALB. (e) Correlation of IL-8 and Hb. (f) correlation of IL-8 and PA. (g) correlation of IL-10 and ALB. (f) Correlation of IL-10 and Hb. (i) correlation of IL-10 and PA. Albumin, ALB; Hemoglobin, Hb; Prealbumin, PA.

As shown in Figure 4, the research group had lower ALB, Hb, and PA than the control group (P<0.05). Pearson correlation coefficient showed a negative association of IL-8 with ALB, Hb, and PA, while a positive association of IL-10 with them (P<0.05), indicating the presence of a correlation of IL-8 and IL-10 with the nutritional status of COPD patients.

### IL-8 was reduced after treatment, while IL-10 was elevated

**Figure 5:**
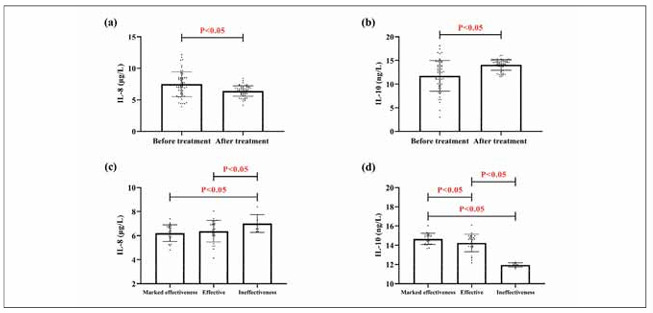
Association of IL-8 and IL-10 with clinical efficacy (a) comparison of IL-8 before and after treatment. (b) Comparison of IL-10 before and after treatment. (c) Comparison of IL-8 with different efficacies. (d) Comparison of IL-10 with different efficacies.

As shown in Figure 5, IL-8 in the research group decreased while IL-10 increased after treatment (P<0.05). The clinical efficacy was assessed as marked effectiveness in 22 cases, effectiveness in 26 cases, and ineffectiveness in 8 cases. Through comparison, we found that IL-8 was lower in patients with marked effectiveness and effectiveness than in patients with ineffectiveness (P<0.05); however, patients with markedly effective treatment showed the highest IL-10 levels among the three groups, followed by those with effective treatment, while patients with ineffective treatment showed the lowest levels (P<0.05).

### IL-8 and IL-10 show excellent diagnostic value for prognostic recurrence

**Figure 6:**
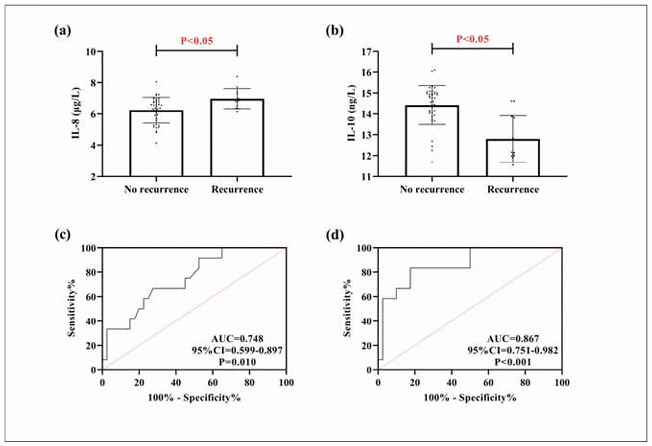
Association of IL-8 and IL-10 with prognostic recurrence. (a) comparison of prognostic recurrent and non-recurrent IL-8. (b) comparison of prognostic recurrent and non-recurrent IL-8. (c) ROC curve for IL-8 diagnosis of prognostic recurrence. (d) ROC curve for IL-8 diagnosis of prognostic recurrence. Receiver operating characteristic, ROC.

During the follow-up of prognosis, 52 cases in the research group were successfully followed up, of which 12 cases had COPD recurrence. As shown in Figure 6, the recurrent patients had higher IL-8 levels and lower IL-10 levels than non-recurrent patients (P<0.05). ROC curves revealed that the diagnostic sensitivity of IL-8 (cut-off>6.33 μg/L) and IL-10 (cut-off<13.88 ng/L) for prognostic recurrence in COPD patients was 91.67% and 83.33%, and the specificity was 47.50% and 82.50%, respectively (P<0.05).

## Discussion

In this study, we found that IL-8 and IL-10 are useful not only for evaluating the occurrence of sleep disorders in COPD patients but also closely related to the progression and prognosis of COPD, which can provide critical clinical references and guidance.

First, we compared the differences in IL-8 and IL-10 between the research and control groups. We found their elevated levels in the research group compared with the control group, suggesting that the two may be involved in the occurrence and development of COPD, consistent with the previous research results [12] [13]. According to the subsequent ROC analysis, both IL-8 and IL-10 demonstrated good diagnostic value for the occurrence of COPD, indicating their potential as diagnostic indicators for COPD. In Ding Q et al.’s [14] study, they also considered IL-8 as a potential marker for COPD and asthma in the future [14], which can also support our view. As for IL-10, a recent clinical study by Jacobs M et al. [15] also found a reduction in IL-10 in smokers and COPD patients, re-emphasising the relationship between IL-10 and COPD. In pathological studies related to COPD, it has been clinically shown that during COPD progression, bronchial and alveolar epithelial cells are constantly stimulated by chronic inflammation, leading to their continuous damage and repair, involving lipopolysaccharide-mediated chronic inflammation, the action of inflammatory cells and cytokines, and the destruction of lung structure and function [16]. Inflammatory cells and their secreted cytokines can stimulate the aggregation of neutrophils, macro phages, lymphocytes, and dendritic cells, as well as release various pro-inflammatory ILs, including IL-8, which can disrupt the immune microenvironment [17]. Therefore, we believe that the increase in IL-8 in the research group in this study is precisely due to the above reasons. The imbalance of the immune microenvironment and the activation and release of pro-inflammatory factors are bound to be accompanied by the suppression of anti-inflammatory factors [18]. Hence, the level of anti-inflammatory factors represented by IL-10 naturally decreases. Because of this, we identified a negative association of IL-8 with FEV1 and FVC and a positive correlation of IL-10 with them when we analysed the correlation of IL-8 and IL10 with lung function. It indicates that the more severe the IL-8/IL-10 expression imbalance, the more severe the COPD condition. Similarly, the research group showed a decrease in IL-8 and an increase in IL-10 after treatment, indicating an improvement in the immune microenvironment. The relationship between the two and clinical efficacy also provides new ideas for future clinical evaluation of the development of COPD. In the research of Pimentel et al., IL8 and IL-10 were shown to participate in the development of hepatitis C by mediating patients’ immune responses [19], which once again confirms our view.

In the analysis of sleep disorders, we can also see that IL-8 is elevated and IL-10 is decreased in COPD patients with sleep disorders, which shows that there is also a close correlation between the two and the occurrence of sleep in COPD patients. Tsai SJ et al. [20] showed that the same elevation of IL-8 was accompanied in patients with major depression, which can support our findings. At the same time, the two also showed excellent diagnostic effects on sleep disorders, suggesting that in the future, we can assess the occurrence of sleep disorders by detecting the expression of IL-8 and IL-10 to intervene in treating patients as early as possible. We believe that the mechanism of the involvement of both in sleep disorders in COPD patients is related to the above-mentioned pathological effects on lung function on the one hand. On the other hand, it may be related to limiting the nutritional status of the patients.

Nutritional status is considered one of the essential factors leading to the development of COPD, with over 60% of COPD patients experiencing significant malnutrition [21]. The decrease in nutritional protein levels in the research group compared to the control group in this article also confirms this viewpoint. The loss of nutritional proteins can directly lead to the imbalance of cellular immune function, increased resting energy expenditure, the aggravation of organ function load, and various complications [22]. We also found a close relationship between IL-8 and IL-10 and patients’ nutritional status, highlighting the relationship between them and the development of COPD. Smidowicz et al. also found a potential connection between the IL family and nutritional proteins [23], and the reasons are as follows: (1) COPD causes abnormal absorption function and delayed gastric emptying, which affects nutrient uptake and absorption; (2) COPD controls appetite in the central nervous system, which leads to anorexia and inflammatory factors invading and compressing the lumen, affecting the secretion of gastric acid, pancreatic juice, bile, and digestive enzymes; (3) The immune microenvironment imbalance promotes the accelerated release of inflammatory factors, which can inhibit the maturation of erythroid hematopoietic progenitor cells, destroy normal hemodynamics in the human body, cause circulatory disorders, and promote chronic loss of nutritional proteins. The deficiency of nutrients such as folate, vitamin B12, and iron further leads to red blood cell disorders, forming a vicious circle. Based on the above results, it can be seen that IL-8 and IL-10 not only directly participate in the occurrence of COPD by affecting the human immune microcirculation but also accelerate the progression of COPD by causing malnutrition, indicating their crucial clinical application potential in COPD in the future.

Finally, through prognostic follow-up, we found that IL-8 and IL-10 exhibited excellent evaluation effects on the prognostic recurrence of COPD patients. This suggests that in the future, the dynamic changes of IL-8 and IL-10 can be monitored to evaluate the prognosis and health of patients, and timely intervention can be carried out for patients at risk of recurrence, thus improving the quality of clinical medical services. However, in previous studies, IL-8 and IL-10 were also considered potential for pancreatic cancer, gastric cancer, and other diseases [24] [25], indicating that the specificity of these two still needs to be concerned in the clinical application of COPD. Meanwhile, we need to increase the number of cases and extend the follow-up period for verification.

## Conclusions

To sum up, IL-8 and IL-10 are closely related to the occurrence and development of COPD and directly impact the nutritional status of patients. In the future, the clinical assessment of sleep disorders, clinical efficacy and prognostic recurrence of COPD can be performed by monitoring the levels of IL-8 and IL-10, which can provide a more reliable guarantee for diagnosing and treating patients and their prognosis.

## Dodatak

### Funding

This study was supported by a grant from the Chongqing Natural Science Foundation (General Program) (No: cstc2020jcyj-msxmX0309).

### Data availability

Original data in this study are available from the corresponding author on reasonable requests.

### Authors contribution

Study conception and design: Mei Liang. Data collection: Qianyun Zhou and Xiaofeng Fu. Data analysis and interpretation: Liang Li. Drafting and critical revision of the article: Mei Zhou. All authors read and approved the final manuscript. Mei Liang and Mei Zhou contributed equally to this work as co-first authors.

### Conflict of interest statement

All the authors declare that they have no conflict of interest in this work.

## References

[1] Christenson S A, Smith B M, Bafadhel M, Putcha N (2022). Chronic obstructive pulmonary disease. Lancet.

[2] Ferrera M C, Labaki W W, Han M K (2021). Advances in Chronic Obstructive Pulmonary Disease. Annu Rev Med.

[3] Adrish M, Anand M P, Hanania N A (2022). Phenotypes of Asthma-Chronic Obstructive Pulmonary Disease Overlap. Immunol Allergy Clin North Am.

[4] Hanania N A, O'Donnell D E (2019). Activity-related dyspnea in chronic obstructive pulmonary disease: physical and psychological consequences, unmet needs, and future directions. Int J Chron Obstruct Pulmon Dis.

[5] Tsai S C (2017). Chronic obstructive pulmonary disease and sleep related disorders. Curr Opin Pulm Med.

[6] Angerio A D (2008). Chronic obstructive pulmonary disease and cytokines. Crit Care Nurs Q.

[7] Liu F, Zhang X, Du W, Du J, Chi Y, Sun B, et al (2021). Diagnosis values of IL-6 and IL-8 levels in serum and bronchoalveolar lavage fluid for invasive pulmonary aspergillosis in chronic obstructive pulmonary disease. J Investig Med.

[8] Lin B, Bai L, Wang S, Lin H (2021). The Association of Systemic Interleukin 6 and Interleukin 10 Levels with Sarcopenia in Elderly Patients with Chronic Obstructive Pulmonary Disease. Int J Gen Med.

[9] Jesus F R, Moraes A C S, da Silva I L N, Passos F C, Salles C, Neves M, et al (2024). Analysis of Endocrine and Inflammatory Markers in Preserved Ratio Impaired Spirometry. Med Sci (Basel).

[10] Jeyachandran V, Hurst J R (2022). Advances in chronic obstructive pulmonary disease: management of exacerbations. Br J Hosp Med (Lond).

[11] Guthrie A (2023). Chronic Obstructive Pulmonary Disease Series Part 4: Identifying, Managing, and Preventing Exacerbations. Sr Care Pharm.

[12] Fang Y, Hu D, Li Q, Chen M, Yin C (2024). Influence mechanism of serum free immunoglobulin light chain on pulmonary inflammatory response and serum levels of inflammatory factors in patients with chronic obstructive pulmonary disease. J Med Biochem.

[13] Li X, Li J, Zhang Y, Zhang L (2021). The role of IL-8 in the chronic airway inflammation and its research progress. Lin Chuang Er Bi Y an Hou T ou Jing Wai Ke Za Zhi.

[14] Ding Q, Sun S, Zhang Y, Tang P, Lv C, Ma H, et al (2020). Serum IL-8 and VEGFA are Two Promising Diagnostic Biomarkers of Asthma-COPD Overlap Syndrome. Int J Chron Obstruct Pulmon Dis.

[15] Jacobs M, Verschraegen S, Salhi B, Anckaert J, Mestdagh P, Brusselle G G, et al (2022). IL-10 producing regulatory B cells are decreased in blood from smokers and COPD patients. Respir Res.

[16] Mkorombindo T, Dransfield M T (2022). Pre-chronic obstructive pulmonary disease: a pathophysiologic process or an opinion term?. Curr Opin Pulm Med.

[17] Govoni M, Bassi M, Santoro D, Donegan S, Singh D (2023). Serum IL-8 as a Determinant of Response to Phosphodiesterase-4 Inhibition in Chronic Obstructive Pulmonary Disease. Am J Respir Crit Care Med.

[18] Kubysheva N I, Postnikova L B, Soodaeva S K, Novikov D V, Eliseeva T I, Novikov V V, et al (2022). Comparative Study of the Levels of IL-1b, IL-4, IL-8, TNFa, and IFNg in Stable Course and Exacerbation of Chronic Obstructive Pulmonary Disease of Varying Severity. Bull Exp Biol Med.

[19] Pimentel J P, Chaves D G, Araújo A R, de Araújo E M, da Silva Fraporti L, Neves W L, et al (2013). Anti-inflammatory/regulatory cytokine microenvironment mediated by IL-4 and IL-10 coordinates the immune response in hemophilia A patients infected chronically with Hepatitis C virus. J Med Virol.

[20] Tsai S J (2021). Role of interleukin 8 in depression and other psychiatric disorders. Prog Neuropsychopharmacol Biol Psychiatry.

[21] Rôlo Silvestre C, Dias Domingues T, Mateus L, Cavaco M, Nunes A, Cordeiro R, et al (2022). The Nutritional Status of Chronic Obstructive Pulmonary Disease Exacerbators. Can Respir J.

[22] Kaluźniak-Szymanowska A, Krzymińska-Siemaszko R, Deskur-Śmielecka E, Lewandowicz M, Kaczmarek B, Wieczorowska-Tobis K (2021). Malnutrition, Sarcopenia, and Malnutrition-Sarcopenia Syndrome in Older Adults with COPD. Nutrients.

[23] Smidowicz A, Regula J (2015). Effect of nutritional status and dietary patterns on human serum C-reactive protein and interleukin-6 concentrations. Adv Nutr.

[24] Feng L, Qi Q, Wang P, Chen H, Chen Z, Meng Z, et al (2018). Serum levels of IL-6, IL-8, and IL-10 are indicators of prognosis in pancreatic cancer. J Int Med Res.

[25] Liu J, Mao Y, Mao C, Wang D, Dong L, Zhu W (2024). An On-Treatment Decreased Trend of Serum IL-6 and IL-8 as Predictive Markers Quickly Reflects Short-Term Efficacy of PD-1 Blockade Immunochemotherapy in Patients with Advanced Gastric Cancer. J Immunol Res.

